# An introduction to health technology assessment and health economic evaluation: an online self-learning course

**DOI:** 10.1007/s12471-023-01777-0

**Published:** 2023-05-12

**Authors:** Isabell Wiethoff, Silvia M. A. A. Evers, Michelle Michels, Mickaël Hiligsmann

**Affiliations:** 1 Department of Health Services Research, Care and Public Health Research Institute (CAPHRI), Maastricht University, Maastricht, The Netherlands; 2 Centre for Economic Evaluation and Machine Learning, Trimbos Institute, Netherlands Institute of Mental Health and Addiction, Utrecht, The Netherlands; 3 Department of Cardiology, Thoraxcenter, Erasmus MC Rotterdam, Rotterdam, The Netherlands

**Keywords:** Health technology assessment, Economic evaluations, Cardiovascular disease, Resource use, Quality-adjusted life years, Health economics

## Abstract

Cardiovascular diseases impose an enormous burden on patients and society. New health technologies promise to lower this burden; however, novel treatments often come at a high cost. In the Netherlands, health technology assessment (HTA) is increasingly being used to inform policy bodies about the optimal distribution of scarce healthcare resources and to guide decision-making about financing and reimbursement. In particular, economic evaluations, as one pillar of HTA, are frequently used to compare the costs and effects of different interventions. This paper aims to define HTA and its relevance to healthcare policy as well as providing a comprehensive overview of the methodology of economic evaluations targeting health professionals and researchers with limited prior knowledge of this subject. Accordingly, different types of economic evaluations are introduced, together with their respective costs and outcomes. Further, the results of economic evaluations are explained, along with techniques for performing them and methods for coping with uncertainty. In addition to this paper-based learning format, each chapter is complemented by a video lecture with further information and practical examples, helping to better understand and analyse health economic studies.

**Supplementary Information:**

This paper is based on an online course, which offers a video-based learning opportunity in addition to the present paper-based format. The corresponding videos and the course material are available online (10.1007/s12471-023-01777-0).

## Introduction

Cardiovascular diseases (CVDs) remain one of the leading causes of morbidity and death, placing an enormous clinical and financial burden on patients and society [1–3]. In the Netherlands, CVDs affected roughly 1.7 million people in 2020, generating overall cardiovascular-related expenditure of 6.8 billion Euros, which is around 6% of the total amount in Euros spent on healthcare [3]. Although cardiovascular mortality has declined in recent years, the disease burden remains high [2, 3]. Much has already been done to address unmet care needs and to improve patient outcomes. Nowadays, a variety of health technologies, such as pharmacotherapies and medical devices, are available and further treatment options are being developed [4]. Other emerging approaches, such as gene therapy or the use of stem cells in regenerative biology, could soon offer even more promising opportunities to lower the burden of disease. However, innovations in healthcare often come at a high cost, leading to questions focussing on the optimal provision and funding of care [5–7].

While the healthcare needs of the population are rising continuously, available healthcare resources remain scarce. Consequently, decisions about the optimal allocation of resources have to be made [8]. Health technology assessment (HTA) provides a multidisciplinary approach, systematically assessing health technologies in order to maximise the health of the population while improving the efficiency of the healthcare system. HTA can provide a useful basis for providing information about opportunities for financing and organising care [8].

**Fig. 1:**
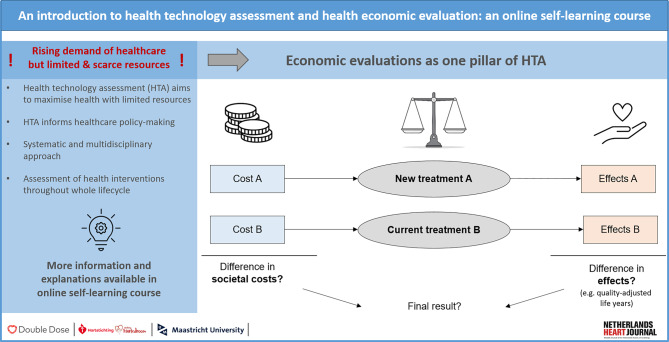
Infographic

This explanatory paper highlights the relevance of HTA in (Dutch) policy-making and provides a methodological overview of economic evaluations (EEs), their different types and respective cost and outcome units (Fig. 1). Approaches for identifying, measuring and valuating these costs and outcomes are further introduced. The last sections explain the results of EEs, techniques for performing EEs and methods for coping with uncertainty. Since this paper is connected to an online self-learning course, each of the following sections is linked to a video lecture (Video 1), which is accessible via the course manual published in the Electronic Supplementary Material.

## Introduction to HTA

HTA (Video 2) is playing an increasingly important role in reimbursement, pricing and funding decisions across the world [9]. In 2020, O’Rourke et al. defined HTA as ‘a multidisciplinary process that uses explicit methods to determine the value of a health technology at different points in its lifecycle. The purpose is to inform decision-making in order to promote an equitable, efficient, and high-quality health system’ [10]. The word ‘technology’ refers to any type of health-promotion intervention, making HTA applicable to pharmaceuticals as well as to medical devices, vaccines, public health programmes etc. [10]. Since HTA can be applied throughout the whole life cycle of technologies, those still under development can be assessed with early HTA to evaluate the potential value of the innovation and to optimise the ongoing development process [10, 11].

Once a technology has passed through the phases of clinical development, it has to undergo a centralised marketing authorisation procedure performed by the European Medicines Agency. This procedure vets new interventions with regard to safety, quality and efficacy [12]. After approval has been granted, pricing and reimbursement negotiations take place at a national level [12]. Here, depending on the country, additional criteria such as effectiveness, budget impact, disease severity, quality of evidence and, in particular, cost-effectiveness are taken into account [10, 12, 13]. For the Netherlands, the most important criteria for reimbursement decisions are the effectiveness, necessity, cost-effectiveness and feasibility of an intervention. Based on the critical appraisal of these criteria, the National Healthcare Institute (*Zorginstituut*) recommends whether a new technology should be added to the basic insurance package or not [14]. However, it is important to note that the use of HTA within this procedure is more applicable to pharmaceuticals than to public health interventions. This appears to be due to more stringent reimbursement policies for pharmaceuticals and methodological challenges in the assessment of non-pharmaceuticals caused by differences in their product characteristics, nature and scope [14].

To provide a reliable basis for decision-making, HTA uses state-of-the-art methods to gather the best available evidence [10]. Commonly used methods in HTA are systematic reviews, meta-analyses, burden-of-disease and cost-of-illness studies, EEs and budget impact analyses [9]. While budget impact analyses focus on the affordability of health technologies, EEs provide information about value for money, i.e. whether the additional benefit of the intervention is worth the additional costs [15].

## Economic evaluations

As one pillar of HTA, EEs are increasingly being used to inform decision-makers about the most efficient allocation of healthcare resources, by comparing the costs and effects of different interventions [16]. Full EEs are especially interesting, as they compare at least two interventions in terms of both costs and consequences and thus provide a full picture of the problem at hand [15]. There are four different types of full EEs, namely cost-minimisation analyses (CMAs), cost-benefit analyses (CBAs), cost-utility analyses (CUAs) and cost-effectiveness analyses (CEAs); these differ in their approach to measuring and valuing outcomes [8, 15].

### Costing

Three steps are necessary to estimate cost outcomes: the identification of relevant costs, their measurement and their valuation in monetary units (Video 3).

#### Identification

**Fig. 2:**
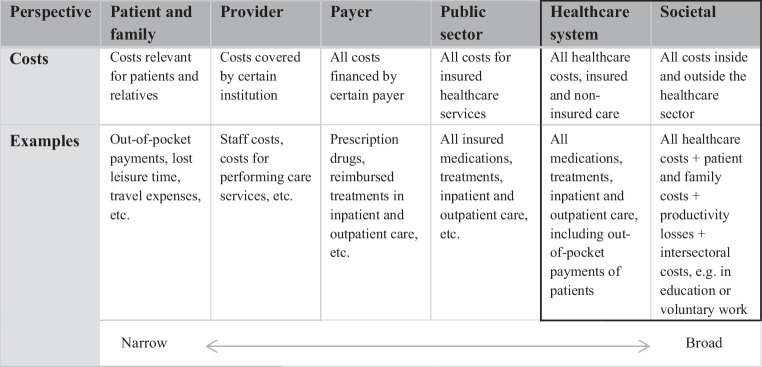
Overview of cost types and perspectives

Drummond et al. distinguished four cost types: (1) healthcare costs, e.g. for treatments, medications, physician visits, etc., (2) patient and family costs, e.g. for out-of-pocket payments, travel expenses and lost leisure time, (3) productivity losses owing to absenteeism and presenteeism and (4) intersectoral costs, i.e. intervention-related costs that might affect other sectors beyond healthcare [15]. The selected perspective of an EE determines which costs are relevant for inclusion [8, 15]. EEs conducted from a societal perspective, as recommended in Dutch guidelines, need to include all four cost types, also called societal costs [17]. Other countries might recommend evaluations performed from the perspective of the healthcare system, which would include costs solely relevant to the healthcare system itself [18]. Narrower perspectives, such as those of the public sector, payer, provider or patient and family, exist; these consider only costs for insured healthcare services or only those costs relevant for a specific patient group or institution [19, 20]. Fig. 2 provides an overview of the different perspectives with the corresponding relevant costs.

#### Measurement

To estimate healthcare costs, usually the patient’s use of healthcare services is measured first. This can be done either top-down, by using aggregated data like databases, or bottom-up at an individual level by using cost diaries or questionnaires, such as the medical consumption questionnaire (iMCQ) [19, 21]. Often, broader non-healthcare costs, such as productivity losses or costs in other sectors, are not included in databases [19]. Those costs can be quantified with self-reported patient data collected via questionnaires, such as the productivity cost questionnaire (iPCQ) [21].

#### Valuation

Once data on patient resource use are at hand, unit costs must be assigned to each of these volumes. For Dutch studies, the Dutch costing manual is recommended for this step [17, 22]. This costing tool entails standard prices for various types of medical care, but also average values for travel expenses, lost leisure time and wages needed to quantify broader cost types, such as patient and family costs and productivity losses [22]. For the calculation of long-term productivity losses, Dutch guidelines recommend the friction cost method [17]. This approach considers the time it takes to replace a worker who is ill, which is called the ‘friction period’ [17, 22]. Another method is the human capital approach, which counts all lost working hours until retirement [20]. If the time horizon in which costs are measured exceeds 1 year, costs must be discounted and adjusted for inflation [17]. For the inflation adjustment, the consumer price index as published by the Dutch Central Agency for Statistics (CBS) can be used to transform costs for a certain reference year [23].

### Outcomes

#### Types of outcomes

**Fig. 3:**
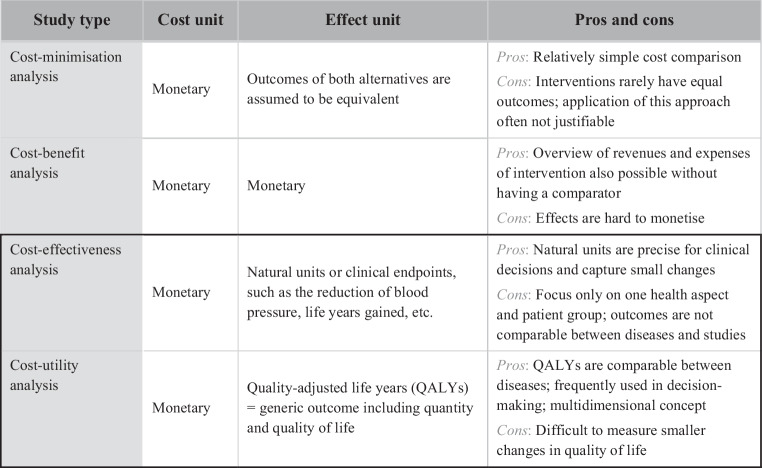
Overview of economic evaluations with corresponding outcomes based on Drummond et al. [15]

While costs are monetised across all four EEs, units representing health effects can vary (Video 4). CMAs assume that interventions are equal regarding health outcomes and select the cheapest option [8]. In CBAs, health effects are like costs valued in monetary units in order to calculate a net benefit ratio [15]. CEAs use clinical outcomes, e.g. potential improvements in blood pressure, left ventricular volumes, New York Heart Association classification, etc. [15]. CUAs use quality-adjusted life years (QALYs) as the outcome; QALYs combine gains in quality and length of life through an intervention in one single measure [15]. Quality of life (QoL) is a multidimensional concept covering various health dimensions, such as the patient’s physical, mental and social well-being. As clinical outcomes often fail to incorporate the patient’s perception of health problems, QoL is often preferred, as it is a more holistic outcome that provides a better understanding of the patient’s well-being and is hence more relevant to the patients themselves [24]. In healthcare, the assumptions of CMAs (equal health outcomes) and CBAs (monetised health benefits) are often inappropriate or difficult to establish. Consequently, CEAs and CUAs are the most frequently performed EEs, and in many countries, including the Netherlands, the recommended types of EEs [9, 17]. An overview of the different types of EEs with their corresponding outcome measures and respective pros and cons is shown in Fig. 3.

#### Measurement

Clinical outcomes are usually derived from trials. For the measurement of QoL, either standardised and generic instruments, such as the EQ-5D or the SF-36 questionnaires, or disease-specific instruments, such as the Kansas City Cardiomyopathy Questionnaire (KCCQ) can be used [24, 25]. Generic questionnaires are usually available and validated in multiple languages and enable the comparison of QoL outcomes between different diseases. Outcomes measured by disease-specific questionnaires cannot be further compared with those of other diseases; however, as they are tailored to a certain disease they might capture more sensitive nuances in the changes of the patient’s QoL [24].

#### Valuation

Generic QoL can be expressed in utilities, with a score between zero (worst health state or death) and one (perfect health), representing an individual’s preference for a given health state [15]. For example, the EQ-5D-5L comprises five health dimensions, each with five answer options, resulting in 3215 possible health states [26]. Corresponding utility scores can be assigned to each health state [26]. These utility scores can be derived from so-called social tariffs, which are utility scores obtained from a reference dataset of the general population [26]. Thereafter, QALYs can be calculated by multiplying the utility score of a health state by the life years gained [15]. One QALY represents 1 year spent in perfect health [15]. Like cost outcomes, effects must be discounted appropriately if the time horizon in which effects are measured exceeds 1 year [17].

### Results of EEs and uncertainty analyses

The cost-effectiveness is assessed by placing the total costs and the outcomes of the compared interventions in relation to each other (Video 5). Therefore, costs and outcomes of the compared alternatives are subtracted respectively, and divided as shown in Eqs. 1 and 2; [8, 15]. If natural units are taken as the outcome, the result is called the incremental cost-effectiveness ratio (ICER):

$$\text{ICER}=\frac{\text{Cost}s_{A}-\text{Cost}s_{B}}{\text{Effect}s_{A}-\text{Effect}s_{B}}=\frac{\Updelta \text{Costs}}{\Updelta \text{Effects}}\left(1\right)$$

If QALYs are chosen the result is called the incremental cost-utility ratio (ICUR) [8, 15]:

$$\text{ICUR}=\frac{\text{Cost}s_{A}-\text{Cost}s_{B}}{\text{QALY}s_{A}-\text{QALY}s_{B}}=\frac{\Updelta \text{Costs}}{\Updelta \text{QALYs}}\;\left(2\right)$$

Both ratios can be interpreted as the additional costs per extra effect unit gained [15]. In order to interpret whether an intervention is cost-effective, a threshold defining the willingness to pay (WTP) per additional unit is needed. Since it is not possible to define a threshold for each natural unit taken as the outcome, many countries define a general WTP threshold for one additional QALY gained [15]. In the Netherlands, an intervention is regarded as cost-effective if the ICUR is below a threshold of 20,000–80,000 Euros per QALY gained; the upper threshold is often chosen for severe conditions like cancer [27]. In contrast, CVDs are often considered preventable, although they cause comparable fatal outcomes; this arouses debate about the suitable threshold [28].

**Fig. 4:**
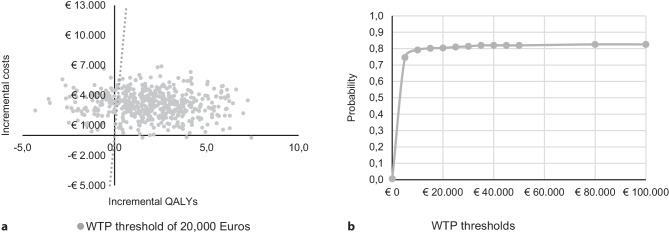
**a**, **b** Fictitious example of a cost-effectiveness plane including Monte Carlo simulations and a cost-effectiveness acceptability curve. The cost-effectiveness plane **a** includes 500 fictitious Monte Carlo simulations and a willingness to pay (*WTP*) line based on the Dutch threshold of 20,000 Euros per quality-adjusted life year gained (*QALY*); the cost-effectiveness acceptability curve **b** represents how many simulations fall below a certain threshold

The results of EEs can be visualised in a cost-effectiveness plane (Fig. 4a). Therefore, incremental outcomes are plotted on the *x*-axis, while incremental costs are plotted on the *y*-axis, allowing the result of the comparison to be shown in one of a total of four different quadrants [16]. Often, new interventions have higher costs but are also more effective than the comparator. Consequently, the ICUR is positive and lies in the upper-right quadrant. Here, policy recommendations are not directly clear and the WTP threshold is used for decision-making. Interventions below the threshold are regarded as cost-effective. Those above the threshold are considered too costly in relation to their additional value. The ICUR of interventions with more effects and lower costs is negative and thus located in the lower-right quadrant. This quadrant indicates that the intervention is highly cost-effective, also called ‘dominant’. Interventions showing higher costs but less effect also have a negative ICUR; however, those lie in the upper-left quadrant and are clearly rejected. In the rare cases that interventions have lower costs and less effect, the ICUR is located in the lower-left quadrant and the resulting recommendations are again unclear. Here the WTP threshold is often extended to this quadrant. However, generally it should be discussed if the use of less effective interventions is reasonable in practice [15].

As EEs depend on various input parameters, which are impacted by different sources of uncertainty, it is crucial to perform sensitivity analyses to draw valid conclusions [16]. Sensitivity analyses explore how results deviate from the base case analysis, which is the result obtained with the preferred set of input data. In deterministic sensitivity analyses, specific parameters are varied individually to test their impact on the results. Probabilistic sensitivity analyses vary multiple, preferably all, parameters simultaneously to test the overall robustness. Monte Carlo simulations are frequently performed; in these procedures an underlying distribution is assigned to an input parameter in order to draw random samples to compute a set of new ICERs/ICURs [15, 16]. These simulations can be presented in the cost-effectiveness plane (Fig. 4a) or, further, in a cost-effectiveness acceptability curve (Fig. 4b) showing the probability of an intervention being cost-effective given a certain threshold [16]. Figure 4 shows both curves for a fictitious example in which the assessed intervention would have a probability of being cost-effective of 80% given a WTP threshold of 20,000 Euros per QALY gained.

### Methods used for EEs

EEs can be performed alongside clinical trials, which directly gather relevant information regarding the patient’s care utilisation or QoL (Video 6). Hence, trial-based EEs usually show a high degree of validity, since all costs and outcomes are measured in the same population. Furthermore, an early assessment of the potential cost-effectiveness is possible. However, in trial-based EEs, the time horizon is limited and the number of comparators is restricted mainly due to practical and ethical reasons [16, 25].

Another approach is the development of decision-analytic models (Video 6), which synthesise all available information from various sources such as literature, databases, registries, etc. and predict health and cost outcomes for a full range of clinical options [16]. Accordingly, a series of potential clinical events, which occur with a certain probability, is defined and linked to corresponding costs and effects [16]. The most frequently used techniques for modelling are decision trees and state transition models, such as Markov models [16]. Their development is subject to country-specific guidelines and requires the use of high-quality data and a multidisciplinary validation of the models’ structure and assumptions [18]. Various checklists provide further instructions on how to adequately perform and critically appraise EEs [29], and to standardise the development of future economic models [30].

## Conclusion

The field of cardiovascular medicine is evolving fast. An ever-growing number of health technologies is becoming available and novel treatment possibilities are being actively explored in clinical research. New interventions have the potential to lower the burden of disease, but also to exert additional financial pressure on already strained healthcare systems. HTA is expected to become increasingly important in guiding funding and reimbursement decisions in the Netherlands and elsewhere. Given the broad spectrum of cardiovascular treatments, EE, as a frequently conducted study type, will play an increasingly important role in the identification of optimal care strategies. This paper offers a concise overview of HTA and the methodology of EEs, including a paper- and video-based learning opportunity, and contributes to a greater awareness of these topics being achieved in the future.

## Supplementary Information

The Electronic Supplementary Material entails the course manual with the links to the video lectures, further reading tips and small exercises (true-false questions) to test the understanding of the topics. The solutions can be found in the appendix of the manual.
