# Supplementary material for: An introduction to health technology assessment and health economic evaluation: an online self-learning course
Source: Neth Heart J. 2023 May 12;31(6):219–25. doi: 10.1007/s12471-023-01777-0 (PMC10188831; doi:10.1007/s12471-023-01777-0)
Supplement: Supplementary file 1 — The Electronic Supplementary Material entails the course manual with the links to the video lectures, further reading tips and small exercises (true-false questions) to test the understanding of the topics. The solutions can be found in the appendix of the manual. [file 12471_2023_1777_MOESM1_ESM.docx]

**Health Technology Assessment (HTA)
Online Course**

Course Manual

March 2022

Contents

[Speakers & Contact 2](#_Toc98244531)

[Summary 3](#_Toc98244532)

[Introduction to the content of the course 4](#_Toc98244533)

[Video 1: Course structure 4](#_Toc98244534)

[Video 2: Introduction into HTA and economic evaluation 5](#_Toc98244535)

[Video 3: Costing 6](#_Toc98244536)

[Video 4: Outcomes 7](#_Toc98244537)

[Video 5: Results and uncertainty analyses 8](#_Toc98244538)

[Video 6: Methods for economic evaluation 9](#_Toc98244539)

[Video 7: Summary lecture 10](#_Toc98244540)

[Video 8: Example I – COI and HRQoL studies 11](#_Toc98244541)

[Video 9: Example II – Economic evaluation 12](#_Toc98244542)

[Video 10: Example III – Systematic literature review 13](#_Toc98244543)

[Video 11: Future Trends in HTA 14](#_Toc98244544)

[Appendix 15](#_Toc98244545)

[Acknowledgement 18](#_Toc98244546)

# **Speakers & Contact**


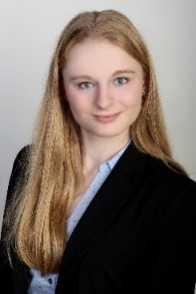
 **Isabell Wiethoff, M.Sc.**

**Contact:** Maastricht University

Department of Health Services Research (HSR)

🕾 +31 (0)43-3882456

E-Mail: [i.wiethoff@maastrichtuniversity.nl](mailto:i.wiethoff@maastrichtuniversity.nl)

Isabell Wiethoff is a PhD student at the HSR department of Maastricht University. After finishing her Bachelor in Health Economics (Bergische Universität Wuppertal, GER), Isabell obtained a Double Master Degree from the universities of Cologne and Maastricht with a special focus on health economics and healthcare policy, innovation and management. The research focus of the PhD lays on the economic consequences and the quality of life impact of inherited cardiomyopathies on patients and families and is part of the Double Dose research consortium.


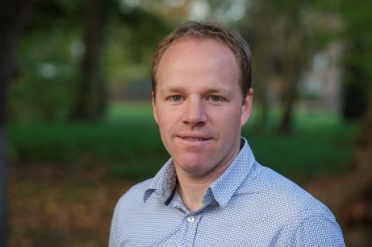
**Mickaël Hiligsmann, PhD**

**Contact:** Maastricht University

Department of Health Services Research (HSR)

🕾 +31 (0)43-3882219

E-Mail: [m.hiligsmann@maastrichtuniversity.nl](mailto:m.hiligsmann@maastrichtuniversity.nl)

Mickaël Hiligsmann is Associate Professor in Health Economics and Health Technology Assessment at the Department of HSR, CAPHRI Care and Public Health Research Institute, Maastricht University (the Netherlands). He holds a PhD in Health, Medicine and Life Sciences from Maastricht University (2015) and a PhD in Medical Sciences (2010), as well as a master in Public Health (2006) and a master in Economics (2003) from the University of Liège. As a researcher, he has more than 15 years of experience in health economic evaluation including cost-effectiveness analyses, decision-analytic modelling, valuation of health care (e.g. discrete-choice experiments, best-worst scaling) and medication adherence. He has been co-promotor of about 20 PhD students, is senior lecturer in HTA and health economic courses and is currently author of about 225 peer-reviewed articles.


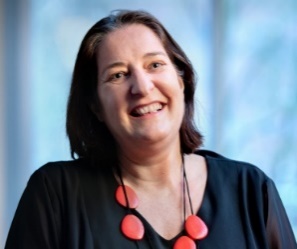
**Prof. Silvia Evers, PhD**

**Contact:** Maastricht University

Department of Health Services Research (HSR)

🕾 +31 (0)43-3881602

E-Mail: [s.evers@maastrichtuniversity.nl](mailto:s.evers@maastrichtuniversity.nl)

Silvia Evers is professor of Public Health Technology Assessment at Maastricht University (UM) and scientific director of the Care and Public Health Research Institute (CAPHRI). Since the early nineties, she has been working as a researcher in the field of Economic Evaluations/Health Technology Assessment at the Institute for Rehabilitation Research, the Maastricht University (Epidemiology; Health Economics; Medical Sociology), the National Institute for Public Health and the Environment (Centre for Care Studies), the University of Amsterdam (Pedagogics), and the University Hospital Maastricht (Neurosurgery). Alongside her research, she has also been working as a legal and policy advisor at the Faculty of Psychology of Maastricht University, where she headed the Research Institute of the Faculty Board.

# **Summary**

**Background:**

Health technology assessment (HTA) of new or existing interventions plays a major role in healthcare decision-making and becomes increasingly important due to ever-growing healthcare costs given limited budgets. To improve the awareness and understanding of health economic related topics, this self-learning HTA course offers several pre-recorded video lectures, which provide a comprehensive overview of various topics in the HTA field. Thereby, the course will mainly focus on economic evaluations and their methods used to assess the cost-effectiveness of an intervention. Next to the pre-recorded video lectures, the course also entails practical examples, further reading tips and small exercises to test the understanding of the topics.

**Objectives:**

This self-learning HTA course aims:

- to provide insights and knowledge about several aspects of HTA with a special focus on economic evaluations.
- to enhance the understanding of methods used in different health economic studies and the interpretation of their results.
- to strengthen the acquired knowledge with practical applications and examples.
- to improve the awareness of health economic related topics.

# **Introduction to the content of the course**

## Video 1: Course structure

The first video of the lecture series provides an introduction into the structure, background and aims of the course. As this course has a self-learning format, it can be followed individually and remotely. Overall, the course offers eleven pre-recorded lectures, each around 15 to 20 minutes long. The lectures can be separated into seven basic lectures, which explain the underlying theoretical concepts of HTA and economic evaluations, and four application lectures, which discuss some example studies to apply the acquired knowledge. As the lectures build on each other, it is advisable to watch the videos in the predefined order. Video seven, the summary lecture, provides a compact overview of all topics covered during the basic lectures and can be re-watched anytime to refresh your HTA knowledge.

**Video Link:**

<https://youtu.be/-T4T9jInVms>

**Basis lectures:**

1. Course structure
2. Introduction into HTA and economic evaluation
3. Costing
4. Outcomes
5. Results and uncertainty analyses
6. Methods for economic evaluation
7. Summary lecture

**Application lectures:**

1. Example I – COI and HRQoL studies
2. Example II – Economic evaluation
3. Example III – Systematic literature review
4. Future trends in HTA

Next to the videos, the course manual provides additional material as well as the sources and further readings. The key source is the book of Drummond et al. (2015), which entails additional information and further examples around economic evaluations. To check the understanding of the topics, small exercises are attached to the manual and can be found at the end of each thematic block. The solutions with the corresponding explanation can be found in the appendix of the course manual.

## Video 2: Introduction into HTA and economic evaluation

The second video provides a general introduction into HTA and its different topics and methodologies. Next to the definition of HTA, also its relevance in healthcare policy and decision-making is emphasized. Moreover, different types of economic evaluations are introduced as one important branch of HTA. A special focus lies on full economic evaluations like cost-minimization analyses (CMA), cost-benefit analyses (CBA), cost-effectiveness analyses (CEA) and cost-utility analyses (CUA). Further, the difference between these types of analyses is discussed by paying attention to the outcome units, which are used for measuring the costs and effects. The knowledge about economic evaluations, which is gained in this video, is the basis for the following lectures.

**Video Link:**

<https://youtu.be/di0v4kwL_xQ>

**Sources & further readings:**

- Drummond, M., et al. (2015). Methods for the economic evaluation of health care programmes. Oxford, Oxford University Press.
- EuNethta (2017). An analysis of HTA and reimbursement procedures in EUnetHTA partner countries: final report.
- European Federation of Pharmaceutical Industries and Associations (EFPIA) (2021). The Pharmaceutical Indistry in Figures. Key data 2021.
- Health Technology Assessment international – HTAi. Video - What is early HTA?
  Link: <https://www.youtube.com/watch?v=1-w9yrg--gU&t=124s>
- Ijzerman, MJ., et al. (2017). "Emerging Use of Early Health Technology Assessment in Medical Product Development: A Scoping Review of the Literature." Pharmacoeconomics 35(7): 727-740.

**True or False?**

1. HTA is performed to reduce healthcare spending and to save money.
2. In a full economic evaluation, a comparison of costs or consequences between two treatments is made.
3. In a cost-benefit analysis, both the costs and consequences are expressed in monetary terms.

## Video 3: Costing

The third video focusses on the cost side of economic evaluations and the cost assessment. Thereby, burden of disease (BoD) and cost-of-illness (COI) studies are introduced as important informative tools, which gather information about the societal and economic impact of health problems. Further, the methodology of COI studies are explained by focusing on the identification, measurement and valuation of costs. Cost are classified into different types according to Drummond (2015). The relevance of each cost type is depending on the chosen perspective of the study. Next, different approaches and instruments for the measurement of the healthcare resource use are introduced whereby the iMCQ and iPCQ cost questionnaires are explained in more detail. Lastly, the Dutch costing tool for the valuation of the resource use and further considerations for the final cost assessment like discounting or inflation adjustment are explained.

**Video Link:**

<https://youtu.be/Nrp4SLmHhB0>

**Sources & further readings:**

- Drummond, M., et al. (2015). Methods for the economic evaluation of health care programmes. Oxford, Oxford University Press.
- Hakkaart-van Roijen L, V. d. L. N., Bouwmans CAM, Kanters T, Tan SS (2015). Methodology of costing research and reference prices for economic evaluations in healthcare, iMTA.
- Institute for Medical Technology Assessment (iMTA). "Questionnaires for the measurement of costs in economic evaluations." from https://www.imta.nl/questionnaires/ [16.12.2021].
- Larg, A. and J. R. Moss (2011). "Cost-of-illness studies: a guide to critical evaluation." Pharmacoeconomics 29(8): 653-671.
- Oostenbrink, J. B., et al. (2002). "Standardisation of Costs: The Dutch Manual for Costing in Economic Evaluations." Pharmacoeconomics 20(7): 443-454.
- Zorginstituut Nederland (2016). “Guideline for economic evaluations in healthcare”. From https://tools.ispor.org/PEguidelines/countrydet.asp?c=22&t=1 [16.12.2021].

**True or False?**

1. The societal perspective is broader than the healthcare perspective.
2. Burden of disease studies capture the economic impact of a certain health problem.
3. In the bottom-up approach, healthcare resources are always measured prospectively and in the top-down approach, always retrospectively.

## Video 4: Outcomes

This video presents different types of outcomes used in economic evaluations. Especially health-related quality of life (HRQoL) is frequently used as it contains several health dimensions of a health problem. Thereby the video explains the difference between generic and disease specific outcomes with their pros and cons. Further, the video explains how HRQoL can be measured by introducing preference-based vs. non-preference based methods. The video will mainly focus on non-preference based methods such as questionnaires like the EQ-5D-5L and the SF-36 as those are commonly used and well known standardized generic instruments. Special attention is paid towards the quality adjusted life year (QALY) outcome, which is a utility score that displays a health state as a number between 0 (worst health) and 1 (perfect health).

**Video Link:**

<https://youtu.be/O36R0o2gVHE>

**Sources & further readings:**

- Drummond, M., et al. (2015). Methods for the economic evaluation of health care programmes. Oxford, Oxford University Press.
- EuroQol Research Foundation (2022). Explaining the EQ-5D in about two-and-a-half-minutes. From https://euroqol.org/eq-5d-instruments/ [28.01.2022].
- Haraldstad, K., et al. (2019). "A systematic review of quality of life research in medicine and health sciences." Quality of Life Research 28(10): 2641-2650.
- Versteegh, M., et al. (2016). "Dutch Tariff for the Five-Level Version of EQ-5D." Value in Health 19(4): 343-352.

**True or False?**

1. Life years gained are an incomplete measure, because they neglect the quality of life dimension.
2. Health has five dimensions, which can be measured with e.g. the EQ-5D-5L.
3. In an economic evaluation, we are comparing the cost-effectiveness of two smoking cessation programs. The primary outcome of this economic evaluation study is the percentage of seven days abstinent people. Thus, this study is a cost-effectiveness study.

## Video 5: Results and uncertainty analyses

The fifth video synthesizes the knowledge of the cost and outcome lectures and explains the concept of incremental analyses as the results of economic evaluations. Thereby, the video differentiates between the incremental cost-effectiveness ratio (ICER) and the incremental cost-utility ratio (ICUR). The latter one uses QALYs as outcome. The ICUR can be interpreted as the additional costs per extra unit of effect gained. For the interpretation of the ICUR, country-specific willingness to pay thresholds are defined, which express the amount of money decision makers are willing to pay per additional QALY. If the ICUR is below the threshold, the intervention is seen as cost-effective. Further, the cost-effectiveness plane and its four quadrants are explained as a common way to display the results of economic evaluations. As economic evaluations are impacted by different kinds of uncertainty, deterministic and probabilistic sensitivity analyses and their methods are further outlined. Special attention is paid on the correct interpretation of graphs used in economic evaluations.

**Video Link:**

<https://youtu.be/fbp3gUyEINI>

**Sources & further readings:**

- Avancena, A. L. V., et al. (2021). "Cost-effectiveness of implantable ventricular assist devices in older children with stable, inotrope-dependent dilated cardiomyopathy." Pediatric Transplantation 25(4).
- Drummond, M., et al. (2015). Methods for the economic evaluation of health care programmes. Oxford, Oxford University Press.
- European Parliament (2015). “Towards a Harmonised EU Assessment of the Added Therapeutic Value of Medicines”.
- Haag, M. B., et al. (2020). "Cost-Effectiveness of Implantable Cardioverter-Defibrillators in Children with Cardiac Conditions Associated with Risk for Sudden Cardiac Death." Pediatric Cardiology 41(7): 1484-1491.
- Wordsworth, S., et al. (2010). "DNA testing for hypertrophic cardiomyopathy: a cost-effectiveness model." European Heart Journal 31(8): 926-935.
- York Health Economics Consortium (2016). “A Glossary of Health Economic Terms.” York. Retrieved from https://yhec.co.uk/resources/glossary/.

**True or False?**

1. A negative ICER means that a strategy is dominant.
2. Most innovations are more effective but also more costly.
3. A new intervention is associated with a QALY gain of 0.2 and a cost increase of €3000 compared to usual care. At a threshold of €20,000, the new intervention is considered cost-effective.

## Video 6: Methods for economic evaluation

Video six goes a step back and explains the different methods used in economic evaluations in order to perform incremental analyses. One way is to perform economic evaluations alongside clinical trials, which directly include instruments for the measurement of costs and outcomes. Another option is to use decision analytic modelling to synthesize all available evidence and to predict the health and cost consequences of an intervention. Therefore, methods like decision trees or state transition models like Markov models are explained in more detail. For both options, advantages and disadvantages are outlined and discussed. Further, the video refers to different guidelines and checklists, which are of major importance for the conduct and reporting of economic evaluations.

**Video Link:**

<https://youtu.be/P6-Awg-HtCw>

**Sources & further readings:**

- Briggs, AH., Claxton K., Sculpher, MJ (2006). Decision modelling for health economic evaluation. Oxford: Oxford University Press.
- Catchpool, M., et al. (2019). "A cost-effectiveness model of genetic testing and periodical clinical screening for the evaluation of families with dilated cardiomyopathy." Genetics in Medicine 21(12): 2815-2822.
- Hakkaart-van Roijen L, V. d. L. N., Bouwmans CAM, Kanters T, Tan SS (2015). Methodology of costing research and reference prices for economic evaluations in healthcare, iMTA.
- Husereau, D., et al. (2022). "Consolidated Health Economic Evaluation Reporting Standards 2022 (CHEERS 2022) Statement: Updated Reporting Guidance for Health Economic Evaluations." Value in Health 25(1): 3-9.
- Leurent, B., et al. (2018). "Missing data in trial-based cost-effectiveness analysis: An incomplete journey." Health Economics 27(6): 1024-1040.
- Zorginstituut Nederland (2016). “Guideline for economic evaluations in healthcare”. From https://tools.ispor.org/PEguidelines/countrydet.asp?c=22&t=1 [16.12.2021].

**True or False?**

1. The first steps in developing a health economic decision model is to conceptualize the decision problem and based on this, the model structure.
2. The use of decision-analytic modelling is often preferred to assess the cost-effectiveness of drugs.
3. A decision tree allows to accommodate changes over time between health states.

## Video 7: Summary lecture

Video seven summarizes the content of the basic lectures and does not introduce new topics. Within 30min, the most important key points out of videos two to six are repeated by providing additional explanations and a further elaboration of relevant terms and concepts. Already familiar content is discussed again in a different wording to present the subjects from a different angle in order to facilitate the memorization of what has been learned.

The purpose of this video is to offer a detailed summary, which can be used to refresh your knowledge of all previous videos. Video seven is thus a compact repetition, which can be watched at a later point of time and re-watched when necessary.

**🡪 Continue with application lectures**

**Video Link:**

<https://youtu.be/UOREgJKUNqQ>

**Sources & further readings:**

None

**Please complete:**

A __________________ compares intervention that have similar consequences.

______________ and ____________________ are examples of decision analytic models.

Economic evaluations looks at the ______________ and ______________ of health interventions.

A _____________ study estimates the health care costs (direct costs) and production losses (indirect costs) of particular disease or risk factor.

In order to perform decision analytic modelling, generally a series of potential _________, each with a certain_________ and the corresponding ______ and ________ need to be defined.

## Video 8: Example I – COI and HRQoL studies

Video eight is the first application lecture, which applies the knowledge out of video three - about the cost assessment, and video four - about the outcome assessment. During this video lecture, the COI study by Jain et al. (2021) and the HRQoL study by Christiaans et al. (2009) are discussed. The COI study estimates the costs and healthcare resource utilization of patients with obstructive hypertrophic cardiomyopathy in the US, while the HRQoL study investigates the quality of life and distress in hypertrophic cardiomyopathy mutation carriers in the Netherlands. Both studies are analyzed regarding their most important study characteristics and their corresponding study results in order to get a feeling how COI or HRQoL studies function. Further the appropriateness of the used methodology and potential limitations are critically assessed.

**Video Link:**

<https://youtu.be/YuZC5Sclc0M>

**Sources:**

- Christiaans I., et al. (2009). Quality of life and psychological distress in hypertrophic cardiomyopathy mutation carriers: A crosssectional cohort study. American journal of medical genetics. Part A 149A:602–612.
- Jain, S. S., et al. (2021). Clinical and economic burden of obstructive hypertrophic cardiomyopathy in the United States. Journal of Medical Economics, 24(1), 1115-1123. https://doi.org/10.1080/13696998.2021.1978242

## Video 9: Example II – Economic evaluation

Video 9 presents and discusses an example of an economic evaluation. The Australian cost-effectiveness study by Catchpool et al. (2019) which compares genetic testing with periodical clinical screening in families with dilated cardiomyopathy was selected. The analysis of the study starts with a description of the most important study characteristics like the research question, the perspective, the corresponding willingness to pay threshold and the selected time horizon. The major focus lies on the explanation of the methods used in this economic evaluation, which is a decision tree and a state transition model. Further, the ICUR is exemplified by showing the calculation and by interpreting the result. Lastly, the results of the sensitivity analyses are shown, interpreted and discussed in order to draw a final conclusion of the decision problem.

**Video Link:**

<https://youtu.be/IYkc3AQkwKk>

**Sources:**

- Catchpool, M., et al. (2019). "A cost-effectiveness model of genetic testing and periodical clinical screening for the evaluation of families with dilated cardiomyopathy." Genetics in Medicine 21(12): 2815-2822.

## Video 10: Example III – Systematic literature review

Video 10 presents the findings of a recent systematic literature review, which provides an overview of currently available COI studies and economic evaluations in dilated and hypertrophic cardiomyopathies. In this video, first the relevance and importance of systematic reviews for healthcare decision-making is highlighted. Further, the methodology of a systematic review is briefly explained. The key focus of the video lies on the reporting of identified studies and their study characteristics, like the patient population, the intervention, the country, the perspective as well as the quality assessment of each study. The results of the identified economic evaluations are synthesized in a cost-effectiveness plane, which applies the knowledge previous video lectures. In addition, the cost-effectiveness for interventions are discussed in more detail for different subgroups. Recommendations for future research are drawn, by providing an overview of the unraveled literature gaps.

**Video Link:**

<https://youtu.be/1NN_EkmPFPc>

**Sources:**

- Wiethoff et al. (in submission). “A Systematic Literature Review of Economic Evaluations and Cost-of-illness Studies of Inherited Cardiomyopathies”.

## Video 11: Future Trends in HTA

The last video of the lecture series deals with future trends in HTA. The purpose of this video is to introduce some current developments of HTA by providing examples of the most important tendencies within this field. Therefore, five key topics are selected and discussed: (1) the industry engagement and involvement, (2) real-world evidence, (3) patient, carer, and citizen involvement, (4) patient and carer preferences, and (5) emerging elements of value.

**Video Link:**

<https://youtu.be/oyOJM-QjhLU>

**Sources & further readings:**

- The Economist. Intelligence Unit. (2021). “New developments in HTA: Evolution not revolution in Health Technology Assessment”. Whitepaper retrieved from https://impact.economist.com/perspectives/healthcare/new-developments-hta-evolution-not-revolution-health-technology-assessment#:~:text=from%20Alan%20Lovell-,New%20developments%20in%20HTA%3A%20Evolution%20not%20revolution%20in%20Health%20Technology,seen%20in%20the%20near%20future [14-03-2022].

# **Appendix**

**Solutions exercises:**

**Video 2: Introduction into HTA and Economic Evaluation**

1. HTA is performed to reduce healthcare spending and to save money.

   Answer: False! HTA is performed to gain maximal health for the population for the available resources. As new innovations are often very costly, it is more about improving efficiency, and not about saving money only.
2. In a full economic evaluation a comparison of costs or consequences between two treatments is made.

   Answer: False! In a full economic evaluation, a comparison of costs and consequences between two treatments is made.
3. In a cost-benefit analysis both the costs and consequences are expressed in monetary terms.

   Answer: True!

**Video 3: Costing**

1. The societal perspective is broader than healthcare perspective.

   Answer: True! The societal perspective also includes productivity losses and cost in other sectors that are not captured in the healthcare perspective.
2. Burden of disease studies capture the economic impact of a certain health problem.

   Answer: False! Burden of disease studies have a broader view. They look at the impact, which one or more diseases will have on a certain country or group of countries in a certain year in terms of different epidemiological measures like prevalence, incidence, mortality etc., quality of life and the economic burden. Cost-of-illness studies, however, they only focus on the economic impact a disease might has.
3. In the bottom-up approach, healthcare resources are always measured prospectively and in the top-down approach, always retrospectively.

   Answer: False! This could be the case. However, in bottom-up approaches, cost-questionnaires can be used, which measure the healthcare resources a patient had during e.g. the last three month. Thus, this is a retrospective measure.

**Video 4: Outcomes**

1. Life years gained are an incomplete measure, because they neglect the quality of life dimension.

   Answer: True! Life years gained only consider the quantity but not the quality of life and are thus incomplete measures.
2. Health has five dimensions, which can be measured with e.g. the EQ-5D-5L questionnaire.

   Answer: False! Health has not necessarily five dimensions. The EQ-5D-5L is one example of an commonly used questionnaire which measures health in five different dimensions, however, the SF-36 for example measures health in eight dimensions. Important to know is that health is a multidimensional concept.
3. In an economic evaluation, we are comparing the cost-effectiveness of two smoking cessation programs. The primary outcome of this economic evaluation study is the percentage of seven days abstinent people. Thus, this study is a cost-effectiveness study.

   Answer: True! This is an example of a cost-effectiveness study, since the outcome chosen here is a natural unit, but no utility score.

**Video 5: Results and Uncertainty Analysis**

1. A negative ICER means that a strategy is dominant.

   Answer: False! A negative ICER could mean that a strategy is dominant. It would be located in the right lower quadrant of a cost-effectiveness plane, respectively. However, pay attention, also an intervention that is more costly and has lower effects has a negative ICER. This ICER would be located in the left upper quadrant of a cost-effectiveness plane and would mean the opposite recommendation for the intervention of interest.
2. Most innovations are more effective but also more costly.

   Answer: True! Commonly, innovations are more effective and more expensive, leading to extra total costs.
3. A new intervention is associated with a QALY gain of 0.2 and a cost increase of €3,000 compared to usual care. At a threshold of €20,000, the new intervention should be accepted.

   Answer: True! As the ICUR is calculated by dividing the incremental costs by the incremental QALYs (€3,000 / 0.2), the result is €15,000 per QALY gained, what is below the threshold of €20,000. Thus, the new intervention is regarded as cost-effective and should be recommended.

**Video 6: Methods for Economic Evaluation**

1. The first steps in developing a health economic decision model is to conceptualize the decision problem and based on this, the model structure.

   Answer: True! First, the decision problem and the relevant patient population, interventions, etc. with its boundaries must be defined. Afterwards the structure of the decision model can be developed based on the previous specifications. Thus, the decision model for each decision problem is different and unique.
2. The use of decision-analytic modelling is often preferred to assess the cost-effectiveness of drugs.

   Answer: True! The use of models is often needed to adequately report the long-term consequences of drugs, but a trial-based evaluation could also be relevant depending on disease specificities.
3. A decision tree allows to accommodate changes over time between health states.

Answer: False! A state transition model involves different health states and can handle longer time horizons. With decision trees, the representation of time is not explicitly possible.

**Video 7: Summary**

A cost-minimization analysis compares intervention that have similar consequences.

Decision tree and Markov models are examples of decision analytic models.

Economic evaluations looks at the costs and outcomes of health interventions.

A cost of illness study estimates the health care costs (direct costs) and production losses (indirect costs) of particular disease or risk factor.

In order to perform decision analytic modelling, generally a series of potential events, each with a certain probability and the corresponding costs and outcomes need to be defined.

# **Acknowledgement**

**Many thanks for following the HTA course!**

As the speakers and organizers of the course, we would like to thank you for listening to the videos about health technology assessment and economic evaluations and for your time given to the material. We hope that you enjoyed the learning experience and that you were also able to broaden your horizon by getting a more comprehensive and holistic view on your research activities.

On behalf of

Silvia Evers, Mickaël Hiligsmann and Isabell Wiethoff
